# A neural strategy for the inference of SH3 domain-peptide interaction specificity

**DOI:** 10.1186/1471-2105-6-S4-S13

**Published:** 2005-12-01

**Authors:** Enrico Ferraro, Allegra Via, Gabriele Ausiello, Manuela Helmer-Citterich

**Affiliations:** 1 Centre for Molecular Bioinformatics, Department of Biology, University of Tor Vergata, Rome, Italy

## Abstract

**Background:**

The SH3 domain family is one of the most representative and widely studied cases of so-called Peptide Recognition Modules (PRM). The polyproline II motif PxxP that generally characterizes its ligands does not reflect the complex interaction spectrum of the over 1500 different SH3 domains, and the requirement of a more refined knowledge of their specificity implies the setting up of appropriate experimental and theoretical strategies. Due to the limitations of the current technology for peptide synthesis, several experimental high-throughput approaches have been devised to elucidate protein-protein interaction mechanisms. Such approaches can rely on and take advantage of computational techniques, such as regular expressions or position specific scoring matrices (PSSMs) to pre-process entire proteomes in the search for putative SH3 targets.

In this regard, a reliable inference methodology to be used for reducing the sequence space of putative binding peptides represents a valuable support for molecular and cellular biologists.

**Results:**

Using as benchmark the peptide sequences obtained from *in vitro *binding experiments, we set up a neural network model that performs better than PSSM in the detection of SH3 domain interactors. In particular our model is more precise in its predictions, even if its performance can vary among different SH3 domains and is strongly dependent on the number of binding peptides in the benchmark.

**Conclusion:**

We show that a neural network can be more effective than standard methods in SH3 domain specificity detection. Neural classifiers identify general SH3 domain binders and domain-specific interactors from a PxxP peptide population, provided that there are a sufficient proportion of true positives in the training sets. This capability can also improve peptide selection for library definition in array experiments. Further advances can be achieved, including properly encoded domain sequences and structural information as input for a global neural network.

## Background

In the functional genomic era, one of the major goals among molecular and cellular biologists is the understanding of protein interaction networks. Over the past few years, it has become more and more clear that many interactions occur over short regions, often less than 10 amino acids in length within one protein. This is particularly true for protein-recognition modules (PRM), such as Src homology (SH) 2 and 3 domains, WW domains, phosphotyrosine binding domains (PTB), postsynaptic density/disc-large/ZO1 (PDZ) domains, Eps15 homology (EH) domains, and 14-3-3 proteins that typically recognize linear regions of 3–9 amino acids [1]. SH3 domains are generally 50 to 70 residues long. They usually bind short proline-rich peptide sequences about 10 amino acids long and containing the core PxxP [2-4]. Structural studies of peptide-SH3 complexes have shown that peptide ligands can bind in two orientations with respect to the SH3 domain [5,6]. The peptides, which bind in either an N to C or C to N terminal orientation relative to the SH3 domain, conform to either class I ([RK]xxPxxP) or class II (PxxPx[RK]) motifs, respectively. Individual SH3 domains are supposed to exhibit specific preferences for variations of their binding consensus. With an aim to investigating the problem of SH3 specificity, various experimental strategies have been proposed, some of which consist of high-throughput approaches: libraries of peptides are synthesized and their binding ability is then confirmed by different *in vitro *experiments [1,7-9]. The high-throughput approaches, however, have to function within the limits of the current technology for peptide synthesis. The number of possible short peptides, even in a proteome as simple as the one of *S. cerevisiae*, is in the order of 10^7 ^[7] while domain or protein family databases contain more than 1500 SH3 domains.

In this regard, there is an urgent need to develop reliable computational methods to help restrict the sequence space of putative SH3 domain binders.

Sequence-based methodologies so far developed to scan entire proteomes in search of putative SH3 partners are regular expressions [7], position specific scoring matrices (PSSM), PSSM-based procedures [10,11] and machine learning approaches [12,13].

In this manuscript, we describe a protein sequence-based methodology, which uses neural networks (NN) [14,15] as a predictive tool for the binding specificity of a set of baker's yeast SH3 domains. Previously, other research groups have developed methodologies based on various principles to infer SH3 interactions. Bock and Gough [12] proposed a support vector machine (SVM) learning approach, based on primary structure and the residues' physico-chemical features alone, to predict interactions. Martin *et al. *[13] developed a SVM combining a sequence-based description of proteins with experimental information. Reiss and Schwikowski [16] integrated protein sequence information and observed interactions in a probabilistic model, which describes the likelihood of generating the amino acid sequences of the binding partners.

This work has been organized into two parts: in the first we built two class-specific neural networks (i.e. relying on peptides conforming to class I and class II motifs, respectively), whereas in the second part we developed individual domain-specific NNs.

Our results are promising, especially when the experimental data used to set up the method are abundant and of high quality, and suggest it might be worthwhile applying our approach to other SH3 domains of the same organism, to the SH3 domains of other organisms, and even to the problem of specificity of other peptide-recognition modules. Our strategy is suitable for SH3 domains and other PRMs that bind to simple short peptides: interactors of domains that require more extended binding surfaces cannot be identified by this methodology.

## Results and Discussion

### Class-specific results

High-throughput experimental strategies for the study of SH3 domain-peptide interactions would benefit greatly from computational methods developed for inferring putative SH3 binding partners. In the case of SH3 domains, the sequence space of binding peptides can be huge, and experimental approaches might be extremely long and laborious or even impracticable.

In this work, we propose a machine learning approach to the problem of restricting the sequence space of potential SH3 targets, and we set up a neural network (NN) for the inference of SH3 domain binding peptides. The neural model is trained on encoded peptide sequences extracted from the *S. Cerevisiae *proteome [17], as described in Methods.

Training data are grouped in class I and class II peptides, depending on their binding orientation preference and corresponding to the sequence consensus [RK]xxPxxP and PxxPx[RK], respectively. We built a neural network for each class of peptides, and trained it to recognize class-specific binders. Results were obtained applying the neural networks to a test set that never appeared during the learning phase (see Methods).

Neural network results were compared to PSSMs (Position Specific Scoring Matrix, for review see [18]) results, the latter obtained with matrices built on the neural network training data and tested on the neural network test data. In the comparison we evaluated precision, sensitivity, specificity and correlation of each approach:



Results of the neural models and PSSMs are reported in Table 1. For both class I and class II data, the machine learning method performs better than PSSM. In particular NNs are more sensitive and more specific than PSSMs, displaying 77% and 72% sensitivity for class I and II data, respectively, while PSSMs attain 73% in class I and only 64% in class II. Furthermore, sensitivity and specificity results are supported by the value of the precision, which is over 50% for the network model in both types of classes, while PSSMs achieve this level only in class II (52%). In the procedure of scanning a proteome in the search for SH3 binding peptide candidates to be validated in high-throughput protein-protein interaction experiments, the correct inference of true non-binders is also important. In this regard, the higher level of specificity of NNs with respect to PSSMs, implies that they are more reliable sequence filters. Finally, the evaluation of the correlation coefficient, as a global indicator of performance, shows that NNs have a higher classification power than PSSMs do.

### Domain-specific results

In the second part of the work, we developed a neural model for each available domain belonging to class I and class II, with the aim of identifying putative binding peptides specific for single SH3 domains. Following this idea and the single domain binding information (see Methods), we built six neural networks for class I peptides and five neural networks for class II peptides (one for each domain within class I or class II specificity).

We compared the NNs' results to PSSMs' inferences, following a procedure identical to the one adopted for class I and class II predictors and using the same performance indicators (precision, sensitivity, specificity and correlation, see Table 1).

In the case of domain-specific neural networks, the performance, on average, does not achieve the level of class-specific networks (Table 2). In the case of class I SH3 domains, NNs' results are controversial: the correlation is higher than the one obtained with PSSMs in three cases (Rvs167, Sho1, Yfr024), and lower in the three remaining cases (Boi1, Myo5, Ysc84).

Noticeably, in the case of class II SH3 domains, neural networks perform better than PSSM in almost all cases (Rvs167, Yfr024, Ysc84). Boi2 domain was excluded from the analysis because of the scarcity of positive binding peptides (only six, see Table 3), which makes the use of both a PSSM and a neural network unreliable.

### Discussion

The results of this work highlight that unbalanced data have a relevant role in the machine learning approach, indicating that an adequate number of binders is crucial to reliably train a neural network or to determine an acceptable PSSM.

A lack of performance is particularly clear in the cases of class I domain-specific neural networks and PSSMs (Table 2) characterised by a low number of binding peptides, where complete failure alternates with very low values for the indicators. It is worth noting that, for those cases in which the quantity of binders is higher, the neural networks always perform better than PSSMs. The addition of new experimental data will eventually make it possible to apply, with increased confidence, neural network approaches to SH3 binding specificity inference.

Not only the low percentage of binders in the dataset can generate unreliable predictors. There might be more intrinsic reasons. Indeed, sequence interference between binders and non-binders remains a source of peptide misclassification. The identification of specific interaction motifs for each domain should start form the accurate analysis of false positives and requires experimental validation of data or the enrichment of datasets with true interactors. However, cases of relevant sequence similarity between elements in the binder and in the non-binder datasets represents the strongest reason for of a machine learning approach for the problem of domain specificity. Cases of sequence identity between peptides arose from the selection of meaningful positions in the sequence encoding procedure for neural network application (see Methods). Among these cases only a small fraction involves pairs of peptides belonging to both binders' and non-binders' subsets, mainly observed in class II dataset. We choose to consider their contribution as the noise due to sequence similarity. PSSMs did not suffer from sequence identity since they were estimated on full-length peptides (see Methods). Any attempt at estimating PSSM on 6 or 7-residue peptides produced inconsistent results (data not shown).

Furthermore, peptide library studies have shown that the recognition profiles of SH3 domains are highly overlapping [1,8,9]. Thus the high superposition of the binders' space of different domains might represent a source of noise for domain-specific classification methods, such as an NN. Another important feature consists of the quality of the experimental data used to build an inference method. Finally, it is likely that, integrating peptide with SH3 domain sequence data and, if available, structural data (Ferraro *et al.*, manuscript in preparation), would strongly improve the NNs' performance also in inferring single domain binding peptides.

With a sufficient information supply, the methodology presented in this work can be extended to the detection of binders of the entire set of yeast SH3 domains, including those characterised by a binding consensus different form class I and class II motifs. The same methodology can also be applied to SH3 domains of other organisms and to all those domains, such as PDZ, WW and 14-3-3, that interact with short peptides.

## Conclusion

In this work, we have shown that a machine learning approach is a helpful methodology for the inference of SH3 domain binding partners in entire proteome scanning. Neural networks, used as peptide sequence classifiers, identify SH3 domain binding peptides in yeast with higher sensitivity and precision than standard PSSMs. Provided an adequate proportion of true positives in the training set, a neural network can be a skilled computational aid of high-throughput experimental strategies designed for the study of protein-protein interactions. The enrichment of the benchmark with a higher number of binding peptides and with information also coming from the domain sequence and/or domain-peptide 3D complexes, where available, would further improve the performance of the neural network in identifying putative SH3 binders. This suggests a future scenario in which such expert systems will be able to detect all the binding partners of protein recognition modules.

## Methods

### Peptide datasets

Our dataset consists of 1379 yeast peptide sequences 14 residues long, obtained by scanning the *S. Cerevisiae *proteome [17] with two peptide *consensi *that conform to typical class I (RK]-x-x-P-x-x-P) and class II (P-x-x-P-x-[RK]) motifs. The procedure generated a dataset of 672 and 707 peptides in class I and II, respectively. Binding information was collected from the PepSpot experiments described in [7], selecting only SH3 domains whose binding peptides match class I and/or class II *consensus*. These SH3 domains are Rvs167, Yfr024c, Ysc84, Boi1, Boi2, Sho1 and Myo5 (Table 1). For each class of peptides (I and II) a dataset comprising positive (binders) and negative (non binders) cases was identified (Table 3). Supplementary datasets of binding + non-binding peptides were derived for each single domain: six datasets for class I domains (Boi1, Myo5, Rvs167, Sho1, Yfr024, Ysc84) and five datasets for class II domains (Boi1, Boi2, Rvs167, Yfr024, Ysc84) (see Table 3). Out of the 672 peptides in class I, 88 were identified as binding to at least one SH3 domain of class I, whereas, out of the 707 peptides in class II, 131 were identified as binding to at least one SH3 domain of class II. Subsequently, for each class, sets of binders and non-binders specific for each domain were identified (Table 3). Within each domain-specific dataset, the binders' and non-binders' subsets can contain similar but not identical peptides. Sequence similarity characterises the complexity of the problem of domain specificity inference since quite often sequence motifs are not able to correctly identify domain interactors in the sequence space. This suggests that more complex methodologies are required. Indeed, we decided to consider the possible sequence similarity between binders and non-binders as one of the main difficulties that our approach must resolve.

### Training and test set sampling

In this work, we initially built two class-specific neural networks ((NN), one for class I and one for class II peptides). The first neural network was trained and tested on the class I dataset composed by 88 binders and 584 non-binders, while the second neural network was trained and tested on the class II dataset of peptides (131 binders and 576 non-binders).

Subsequently, we built eleven domain-specific neural networks: six for the class I binding domains (Rvs167, Yfr024c, Ysc84, Boi1, Sho1, Myo5) and five for the class II binding domains (Rvs167, Yfr024c, Ysc84, Boi1, Myo5). A SH3 domain-specific neural network was trained and tested using the dataset of its binding peptides obtained from the corresponding domain binding information (see Table 3).

For each NN, training and test sets of peptides were sampled as follows. The 70% of binders and the 70% of non-binders were assigned to the training set, while the remaining 30% of each type (binders and non binders) was used as the test set.

The sampling procedure is random and was repeated five times for each network, in order to compute an average performance of the models.

Each domain is characterised by a strongly unbalanced dataset in terms of binding and non-binding proportion (see Table 3). The unbalancing forced us to adopt a correction procedure in order to build up effective inference models. The dimension of datasets cannot be reduced without affecting the essential requirement of network complexity. Hence, we decided to replicate the binders in the training set of each class and of each SH3 domain, until an equal proportion was established. This enhanced the relevance of positive cases in the learning phase. The test sets were left unbalanced since they must reflect the real proportions between binders and non-binders.

### Sequence encoding

Peptide sequence information is encoded by the standard orthogonal code [14,15]. This type of encoding assumes that a residue is 'translated' into 20 binary variables. Therefore, a 14-residue peptide sequence corresponds to 280 binary variables. This huge amount of input information implies too many neural network parameters with respect to the number of sequences in the datasets, thus causing overfitting. To overcome this problem, we considered only the *consensus *core of the peptides: based on the well-assessed definition of SH3 binding core [2,4,8], it consists of a 7-residue subsequence for class I peptides and a 6-residue sub-sequence for class II peptides. From both types of peptide cores, we excluded the prolines of the PxxP motif: indeed these prolines are common to both positive and negative cases and, therefore, not informative. This filtering procedure left 5 positions for class I peptides and 4 positions for class II peptides, which were encoded by the standard orthogonal code, giving rise to 100 and 80 binary variables, respectively.

The selection of the peptides' consensus core sometimes generates identical 6 residue peptides (from similar 14 residue long peptides). In a few cases, identical peptides can be found in both binders' and non-binders' datasets. Such cases represent a noise source in the training set, and the neural network have to overcome this problem by the robustness and the complexity of the learning algorithm [14].

### Network architecture

The neural network architecture consists of a single hidden layer, besides the standard input and output layers. The composition of the input and the hidden layers depends on the dimension of the input space and on the size of the training set. Thus, class-specific and domain-specific NNs, which depend on class I data, have 100 input variables and 4 hidden units while those which depend on class II have 80 input variables and 5 hidden units. For both types of neural network the output layer consists of a single unit.

As a control procedure we built and tested a position specific scoring matrix (PSSM). For each SH3 domain considered, a PSSM was obtained from the alignment of the training set binders, and tested on the peptides of the corresponding test set. Peptides 14 residues long were used. Each PSSM was calculated and tested (by the Emboss routines 'prophecy' and 'profit' [19]) five times on the NN randomly generated training and test sets of peptides, in order to compute an averaged performance of the PSSM.

## Authors' contributions

EF designed the study and built, trained and tested the neural networks and PSSMs. AV designed and coordinated the study. EF and AV authored the manuscript. GA collected data and participated in the design of the study, MHC supervised the work and revised the manuscript. All authors read and approved the final manuscript.

## Figures and Tables

**Table 1:** Class-specific network and PSSM results. The comparison shows the substantial improvement of the machine learning method with respect to PSSM. The higher sensitivity and precision of the neural model with respect to the PSSM indicate that the former is able to predict a lower number of false positives. The higher specificity of the neural model also implies a better filtering of non-interacting sequences and a higher performance of the model in the detection of SH3 binders.

Class	Number of Binders	PSSM				NN			
		
		*Prec*	*Sens*	*Spec*	*Corr*	*Prec*	*Sens*	*Spec*	*Corr*
I	88 (13.1%)	40	73	84	0.45	51	77	89	0.56
II	131 (18.5%)	52	64	87	0.47	57	72	88	0.55

**Table 2:** Domain-specific neural network and PSSM results. The application of a domain-specific strategy in the detection of binders reveals the strong effect of the data unbalancing. Class I binding domains have a lower percentage of binders within the datasets and in the corresponding results both PSSM and neural networks display low performances, with no clear benefit in preferring one method to the other. The results of class II binding domains, where a higher percentage of binders (Rvs167, Yfr024, Ysc84) is present, clearly show the prevalence of neural networks. For Boi1 and Boi2 the estimation of PSSM and NN is less significant due to the scarcity of binders.

Class	Domain	Number of Binders	PSSM				NN			
			
			*Prec*	*Sens*	*Spec*	*Corr*	*Prec*	*Sens*	*Spec*	*Corr*
I	BOI1	15 (2.2%)	50	25	99	0.34	4	80	47	0.09
	MYO5	35 (5.2%)	57	67	98	0.60	38	53	97	0.41
	RVS167	19 (2.8%)	0	0	99	-0.01	31	68	96	0.43
	SHO1	37 (5.5%)	70	64	98	0.65	64	84	97	0.71
	YFR024	25 (3.7%)	14	14	97	0.11	25	37	94	0.25
	YSC84	12 (1.8%)	100	33	100	0.57	10	80	81	0.24
II	BOI1	16 (2.3%)	17	50	95	0.27	19	38	97	0.25
	RVS167	44 (6.2%)	53	62	96	0.54	59	77	96	0.65
	YFR024	123 (17.4%)	47	56	87	0.40	56	78	87	0.58
	YSC84	67 (9.5%)	61	55	96	0.54	60	83	94	0.67

**Table 3:** Peptide sequence distributions. Peptides are divided into the two classes of binding orientation (I and II). The peptide proportion is reported in the second column. The third and fourth columns contain the number of binders and non-binders, respectively. The fifth column describes class I and class II SH3 domains, with the corresponding proportions of binders and non-binders listed in the last two columns, respectively. The latter information characterizes the domain-specific datasets used to train and test the corresponding domain-specific neural networks. The percentage of binders (3^rd ^and 6^th ^columns) highlights the critical unbalancing and attains acceptable levels only in the two class-specific datasets and in three class II domains in the domain-specific datasets.

Class	Number of Peptides	Number of Binders	Number of Non-binders	SH3 Domain	Number of Binders (%)	Number of Non-binders (%)
I	672	88 (13.1%)	584 (86.9%)	Rvs167	19 (2.8%)	653 (97.2%)
				Yfr024c	25 (3.7%)	647 (96.3%)
				Ysc84	12 (1.8%)	660 (98.2%)
				Boi1	15 (2.2%)	657 (97.8%)
				Sho1	37 (5.5%)	635 (94.5%)
				Myo5	35 (5.2%)	637 (94.8%)
II	707	131 (18.5%)	576 (81.5%)	Rvs167	44 (6.2%)	663 (93.8%)
				Yfr024c	123 (17.4%)	584 (82.6%)
				Ysc84	67 (9.5%)	640 (90.5%)
				Boi1	16 (2.4%)	691 (97.6%)
				Boi2	6 (0.8%)	701 (99.2%)
